# Origins of an Intrinsic Hippocampal EEG Pattern

**DOI:** 10.1371/journal.pone.0007761

**Published:** 2009-11-11

**Authors:** Christopher S. Rex, Laura L. Colgin, Yousheng Jia, Malcolm Casale, Theodore K. Yanagihara, Maria Debenedetti, Christine M. Gall, Eniko A. Kramar, Gary Lynch

**Affiliations:** 1 Department of Anatomy & Neurobiology, University of California Irvine, Irvine California, United States of America; 2 Department of Psychiatry & Human Behavior, University of California Irvine, Irvine, California, United States of America; 3 Department of Neurobiology & Behavior, University of California Irvine, Irvine, California, United States of America; University of Alberta, Canada

## Abstract

Sharp waves (SPWs) are irregular waves that originate in field CA3 and spread throughout the hippocampus when animals are alert but immobile or as a component of the sleep EEG. The work described here used rat hippocampal slices to investigate the factors that initiate SPWs and govern their frequency. Acute transection of the mossy fibers reduced the amplitude but not the frequency of SPWs, suggesting that activity in the dentate gyrus may enhance, but is not essential for, the CA3 waves. However, selective destruction of the granule cells and mossy fibers by in vivo colchicine injections profoundly depressed SPW frequency. Reducing mossy fiber release with an mGluR2 receptor agonist or enhancing it with forskolin respectively depressed or increased the incidence of SPWs. Collectively, these results indicate that SPWs can be triggered by constitutive release from the mossy fibers. The waves were not followed by large after-hyperpolarizing potentials and their frequency was not strongly affected by blockers of various slow potassium channels. Antagonists of GABA-B mediated IPSCs also had little effect on incidence. It appears from these results that the spacing of SPWs is not dictated by slow potentials. However, modeling work suggests that the frequency and variance of large mEPSCs from the mossy boutons can account for the temporal distribution of the waves. Together, these results indicate that constitutive release from the mossy fiber terminal boutons regulates the incidence of SPWs and their contribution to information processing in hippocampus.

## Introduction

Rhythmic oscillations, a characteristic feature of the cortical telencephalon, are generally thought to be important for synchronizing the activity of neurons within and/or across regions. In the hippocampus, different types of rhythmic patterns emerge in association with specific behaviors, the most widely studied and best-understood of which is theta [1]–[3]. Theta rhythms do not occur spontaneously in hippocampus, although they can be triggered by muscarinic agonists [4], but instead appear to be paced by synchronous firing of cholinergic and GABAergic neurons in the lower brain [5], [6]. There is also evidence for aperiodic waves in hippocampus that are intrinsic in that they do not require external inputs for their generation. Sharp waves (SPWs) are a prominent example of such activity [7]–[9]. These are monophasic potentials of approximately 50–100 ms duration that occur at irregular intervals when animals enter into a state of alert immobility or during the slow-wave portion of sleep. The waves have a mean frequency between 0.5 to 5 Hz and carry a high frequency, low amplitude oscillation (‘ripple’) on their ascending phase [10]–[12]. Recent work strongly suggests that ripples are associated with the discharge of nearby interneurons [13], [14] and are dependent upon gap junctions [15].

The recent discovery that SPWs are generated within appropriately prepared hippocampal slices [16], [17] both confirmed the intrinsic nature of the waves and provided a means for investigating their origins. Initial work directed at the latter issue established that sharp waves are composite (population) EPSCs generated within field CA3, most probably in field CA3b, and are propagated along that region's massive associational system [16]. Strong inhibition is also present during a SPW; this presumably serves to shape the waves and to limit the number of cells they incorporate [15]. Collectively, these observations strongly suggest that individual SPWs occur when small populations of neighboring pyramidal cells are recruited by local associational connections into near-synchronous discharge, resulting in a population event that radiates throughout CA3 and into CA1. In accord with this, SPW generation has been associated with synchronous discharges between pyramidal neurons and interneurons in CA3 [18]. Questions then arise about the events that trigger the spontaneous activity that develops into a SPW. Simultaneous recordings from the dentate gyrus and field CA3 indicate that autonomous population discharges in the former (i.e., ‘dentate waves’ or ‘DWs’) are followed with regularity by large SPWs in the latter [19]. However, SPWs are approximately five times more frequent than DWs [19], indicating that the dentate events either initiate reverberating activity in CA3 or simply boost ongoing spontaneous activity.

The present experiments were intended to directly address the questions of how SPWs are initiated, and why they occur with the frequency that they do. One set of studies examined the waves after afferents to field CA3 from the entorhinal cortex and dentate gyrus had been severed, while others tested for contributions from constitutive release. The results indicate that the mossy fiber terminals both trigger and set the frequency of SPWs, and thereby suggest novel ideas about the functional significance of the singular features associated with these endings.

## Results

### General properties of spontaneous sharp waves in vitro

**Figure 1:**
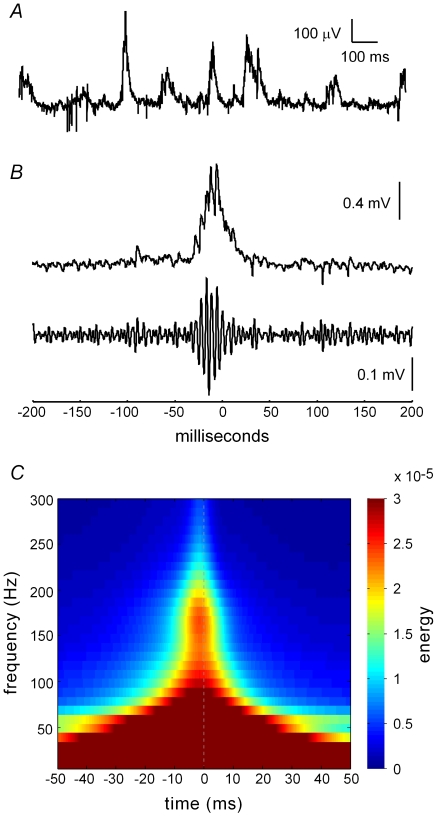
Sharp waves (SPWs) and ripples occur spontaneously in hippocampal slices. ***A***, Typical recording collected from the pyramidal cell layer of field CA3. ***B***, A single SPW is shown unfiltered (top) and after high-pass filtering (bottom); low voltage, high frequency activity (ripples) occurs on the ascending phase of the SPW and decreases quickly after the peak of the wave (0 ms). ***C***, Time frequency representation of sharp wave/ripples. Energy in the ∼150–200 Hz (‘ripple’) range of the signal is maximal in the 10 ms before the peak of the SPW, which occurs in the figure at 0 ms and is indicated by a vertical dotted line.

**Figure 1A** shows a representative trace recorded under baseline conditions from the CA3 pyramidal cell layer. As described previously [16], the primary component of the activity is a positive-going potential in the cell body layer of approximately 50–100 ms duration. Across a set of 13 slices the potentials occurred with an average frequency of 3.3±2.4 Hz (mean ± SD) under baseline conditions. The frequency of the waves within slices varied over time within a ∼0.1–10 Hz range, with a mean coefficient of variation of 69%. Amplitude was also highly variable across slices and from cycle to cycle within slices (data not shown). SPWs in hippocampal slices [15], [16] and in vivo [20] are accompanied by a high frequency oscillation (∼200 Hz ‘ripple’). Ripples were observed on the ascending phase of extracellularly recorded SPWs in CA3 (**Fig. 1B**). Energy of the signal at high frequencies (100 Hz and above) developed during the ∼10 ms period before the SPW reached its peak, and the greatest energy at ripple frequencies (∼200 Hz) was observed just a few ms prior to the maximum amplitude of the SPW (**Fig. 1C**). These observations, collected in interface chambers, are in close agreement with previous studies from this laboratory using simultaneous extracellular and whole cell recordings from submerged slices [21], [22]. It should be noted, however, that optimal conditions for studying SPWs in slices are yet to be determined.

### Contributions of entorhinal cortex and dentate gyrus to CA3 SPWs

It is generally agreed that SPWs are excitatory currents initiated intrinsically within the CA3 region of the hippocampus [7], [12], [16], [23], [24], an area that is highly interconnected via the numerous excitatory axon collaterals from CA3 pyramidal cells [25], [26]. In accord with this, earlier in vitro studies have shown that sharp waves are associated with intracellular EPSCs, accompanied by potent IPSPs, and are eliminated by AMPA receptor antagonists [15], [16].

**Figure 2:**
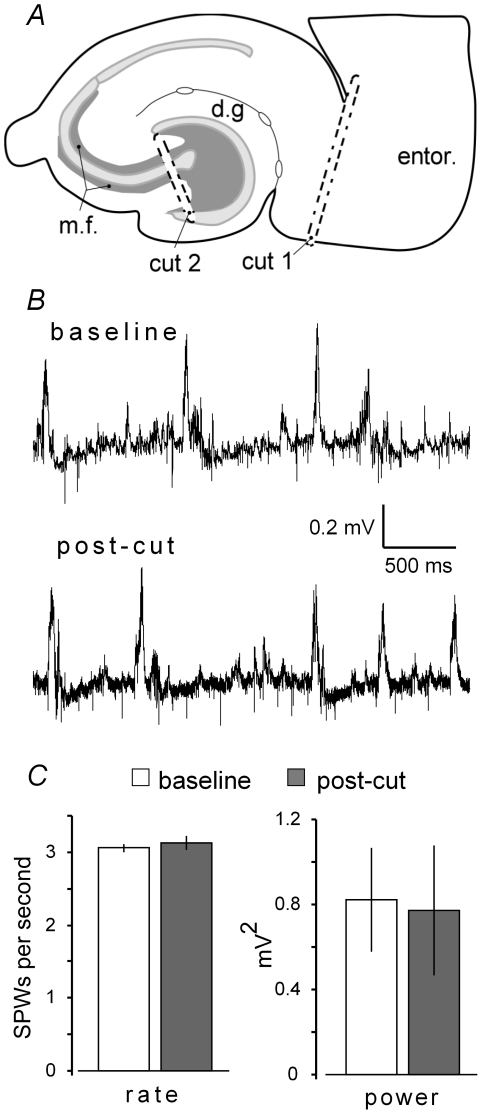
Knife cuts through the projections from entorhinal cortex to hippocampus (perforant path) do not affect SPW activity in field CA3. ***A***, Schematic of a slice from the temporal hippocampus illustrating the location of the knife sections used in the present studies: cut #1 separates the hippocampus from the entorhinal cortex while cut #2 is through the mossy fibers (dark gray) at the entrance to the hilus (m.f: mossy fiber pathway; dg: dentate gyrus). ***B***, Baseline SPWs recorded from CA3 stratum pyramidale (top). Recordings collected at least 30 minutes after severing the perforant path (bottom). ***C***, Mean rate and power (± SEM) of SPWs for a group of five slices prior to and following cuts through the perforant path.

How individual SPWs are triggered is not known and constitutes the experimental question addressed by the present experiments. CA3 pyramidal cells receive excitatory afferents from the dentate gyrus via the mossy fibers, from the entorhinal cortex via the perforant path and temporo-ammonic projections, and from other CA3 pyramidal cells via the massive CA3 commissural-associational projections [27]. Thus, evoked or constitutive activity of any of these pathways could, in principle, initiate an individual SPW. The entire retrohippocampal area is connected to the hippocampus proper in the temporal hippocampal slices in which SPWs are found, and well-developed perforant path responses can be elicited by stimulation of either medial or lateral entorhinal cortex [19]. The absence of intact entorhino-hippocampal connections, and thus of cortically generated oscillatory activity into hippocampus, could explain why SPWs are altogether absent in more rostral hippocampal slices. However, disconnecting the entorhinal cortex (**Fig. 2A**) from the hippocampus with a scalpel cut, in a group of five temporal slices, did not measurably affect ongoing SPW mean rate and power (**Fig. 2B,C**).

**Figure 3:**
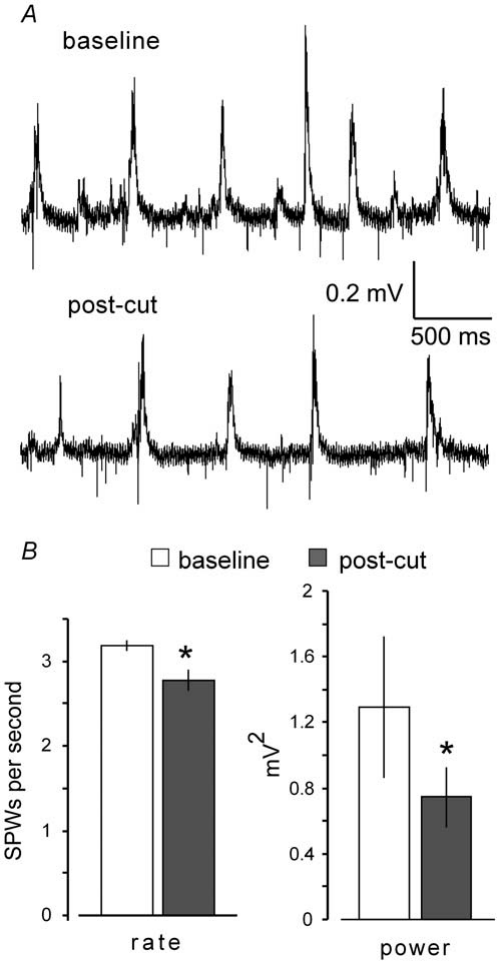
Knife cuts through the connections between the dentate gyrus and field CA3 (mossy fibers) reduce the rate and size of SPWs in the latter region. ***A***, Spontaneous activity collected from CA3 stratum pyramidale prior to (baseline) and at least 30 minutes after cutting the mossy fibers (post-cut). ***B***, Mean rate and power (± SEM) results for a group of five slices (*p<0.02; n = 5 slices).

The dentate gyrus is a likely source of activity that could trigger SPWs in CA3. As noted, past studies have demonstrated that the granule cells generate spontaneous waves and that these precede large SPWs [19], an observation that raises the possibility that a dentate event initiates a cycle of gradually dampening SPWs. This idea was tested by recording spontaneous activity from CA3 stratum (str.) pyramidale before and after scalpel-transections of the mossy fibers at the emergence of this system from the dentate gyrus hilus (**Fig. 3**). The rate of SPW occurrence was reduced by 13±10% following the transection in a group of 9 slices (p<0.05; paired 1-tailed t-test). This small effect could have been due to non-specific disturbances associated with sectioning, although larger cuts of the entorhino-hippocampal projections (see above) had no detectable effects on frequency. The amplitude of individual SPWs appeared smaller after the mossy fiber cut (**Fig. 3A**) and, in accord with this, power spectral analyses indicated that field activity in the SPW frequency range was reduced by about one third post-transection (**Fig. 3B**). These relatively minor effects do not support the hypothesis that spontaneous waves in the dentate gyrus initiate bouts of SPWs but suggest instead that such activity increases ongoing CA3 activity.

### Constitutive release and the genesis of SPWs

Spontaneous glutamate release from the mossy fibers causes ‘giant miniature EPSCs’ recorded in CA3 pyramidal cell somata [28]–[30]. The mean amplitude of these unitary events [28], [30] is an order of magnitude greater than the mean EPSC at commissural-associational synapses in CA3 str. radiatum [31] and perforant path synapses in CA3 str. lacunosum/moleculare [32], [33]. Moreover, the mossy fibers terminate in str. lucidum, a compact layer situated close to the spike initiation zone for CA3 pyramidal cells, and are therefore well-positioned to cause cell firing. As cutting the mossy fibers at the CA3c/hilar boundary would not be expected to block it, constitutive release remains a plausible candidate for the driving force for SPWs. That is to say, the mossy fiber axons could continue to spontaneously release glutamate from their terminals after being severed from the soma and in this manner continue to provide an excitatory trigger for sharp waves.

**Figure 4:**
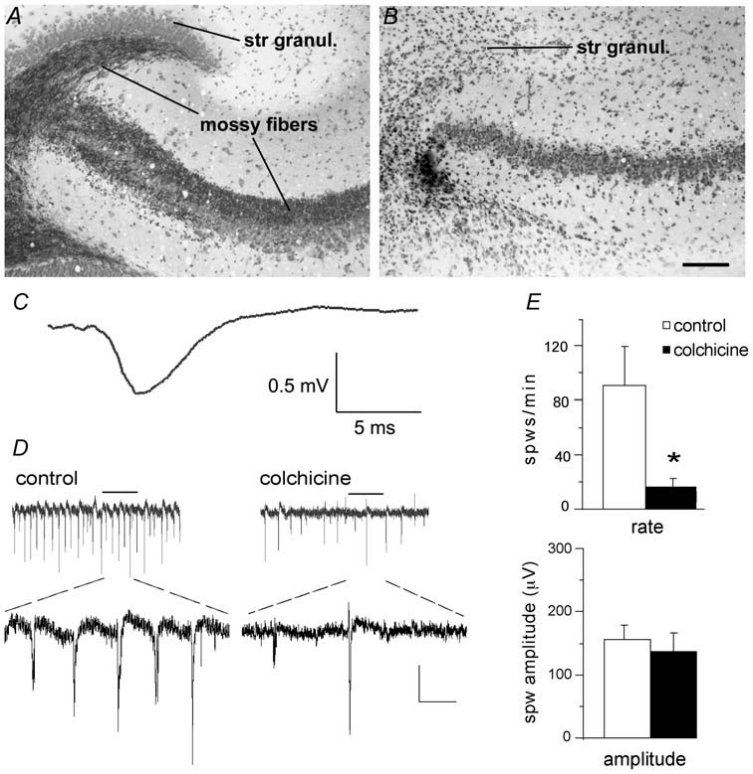
Destruction of the mossy fibers drastically reduces the incidence of SPWs. ***A,B*** *,* Photomicrographs show Timm's- and Nissl- stained sections through slices from an untreated control rat (*A*) and an experimental rat (*B*) that received a local colchicine injection that destroyed the granule cells (stratum granulosum) and mossy fibers while leaving the CA3 pyramidal cells intact (calibration bar  = 150 µm for *A* and *B*). ***C***, Average of 11 EPSPs recorded across 4 min from CA3 stratum radiatum in response to stimulation of CA3 commissural-associational system in a representative slice from the colchicine lesion group. ***D***, SPWs recorded from the apical dendrites of field CA3 in slices from control (left) and colchicine-treated (right) rats. Bottom traces show the indicated segment of top traces magnified to scale. Calibration: 50 µV/500 ms for top traces; 100 µV/1.5 sec for bottom traces. ***E***, The rate of spontaneous SPWs (top) was significantly lower in the colchicine group (n = 4 slices) as compared to controls (n = 3, *p<0.05, 2-tailed t-test), but the average amplitude of SPWs was not significantly affected by the colchicine lesion (bottom).

The above argument predicts that near complete removal of the mossy fibers and consequent loss of spontaneous glutamate release from mossy fiber terminals would produce a profound suppression of SPWs. This was tested by injecting colchicine, a toxin that selectively destroys granule cells [34], [35] and used previously to define the origin of dentate spikes [36], into the temporal hippocampus at least five days prior to preparing slices. The injections removed both wings of the dentate gyrus along with the mossy fibers without substantially changing the pyramidal cells of field CA3 (**Fig. 4A,B**). In agreement with the latter observation, evoked potentials in CA3 str. radiatum, elicited by single pulse stimulation (30 µA, 0.03 ms) of the apical CA3 commissural-associational projections, in slices prepared from colchicine-treated rats, exhibited normal-appearing waveforms (**Fig. 4C**), although response amplitudes (0.68±0.16 mV; n = 4 slices from 4 animals) were below those of slices from naïve animals (0.96±0.19; n = 3 slices from 3 animals). The absence of sizeable effects in CA3 accords with prior reports showing that colchicine-induced destruction of the granule cells does not severely affect theta activity [37] or the activity of CA3 place cells [38]. Despite this, the frequency of SPWs was reduced by more than 80% in slices lacking mossy fibers, as is evident from a comparison of the number of waves per minute in control and experimental slices (**Fig. 4D,E**). Because SPW amplitude is maximal in the apical dendrites both in vivo [7] and in vitro [16], recordings were obtained from str. radiatum (note reversal of sharp wave phase, as expected, compared to earlier figures showing recordings from str. pyramidale). The SPWs that remained were not notably different with regard to their average amplitude (**Fig. 4E**, bottom) from those observed during control conditions, indicating that de-afferentation did not affect the basic machinery for generating and propagating the waves. It appears instead that release from the mossy fibers governs the frequency of the waves while having a lesser influence on their amplitude.

### Agents that increase or decrease release from the mossy fibers

**Figure 5:**
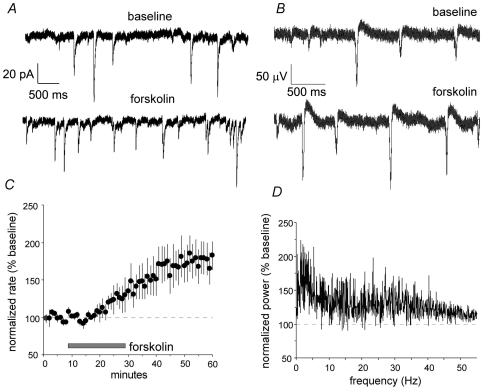
Forskolin increases the frequency of mEPSCs and SPWs. ***A***, Miniature (m) EPSCs recorded from a CA3 pyramidal neuron prior and during the infusion of 10 µM forskolin. ***B***, SPWs recorded from CA3 stratum radiatum prior to (baseline) and 30 min after (bottom) infusing 10 µM forskolin. ***C***, Group data (n = 5 slices) showing the time course over which forskolin increases the rate of SPW occurrence. ***D***, Forskolin has its greatest effect on power of spontaneous oscillations in the SPW (0.1–7.0 Hz) frequency range.

Forskolin, a compound that stimulates the production of cAMP, is reported to increase transmission at mossy fiber synapses [39]. In agreement with this, forskolin (10 µM; 20 min bath infusion) almost doubled the frequency of mEPSCs (0.5±0.3 Hz to 0.89±0.6 Hz; n = 5 cells from 5 slices) recorded from CA3b pyramidal neurons without measurably affecting their amplitudes (23.1±3.3 pA to 20.4±3.8 pA)(**Fig. 5A**). The same treatment had quantitatively similar effects on SPWs in str. radiatum: It doubled their frequency while leaving their amplitude unchanged (**Fig. 5B,C**). Spectral analysis of the same data (**Fig. 5D**) indicated that an increase in power in the 0.1–7 Hz SPW frequency range was accompanied by much smaller changes in the higher frequency bands.

**Figure 6:**
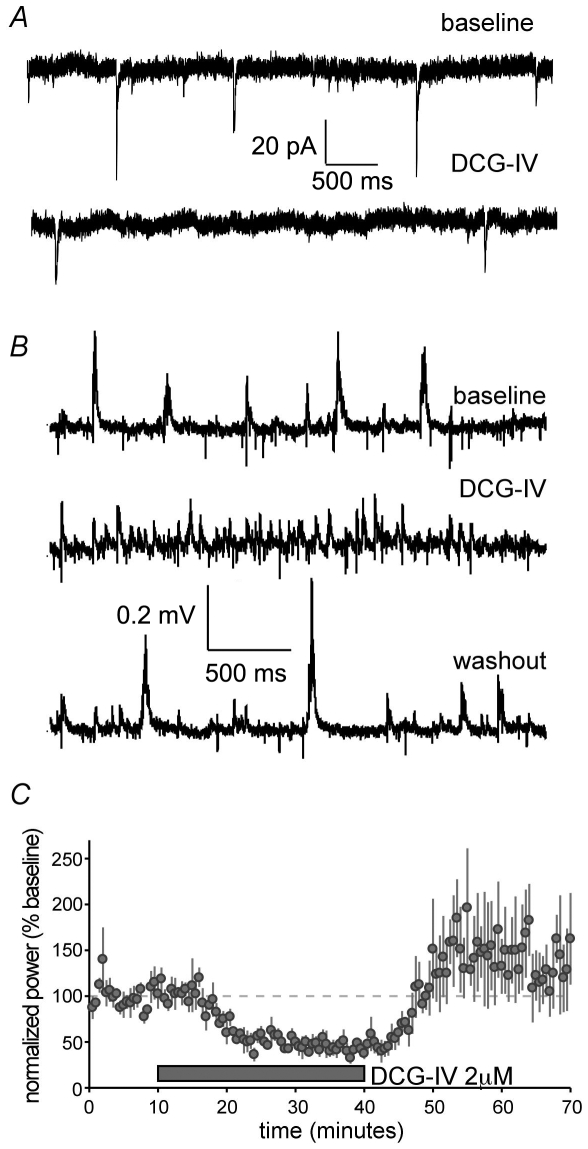
A type 2/3 metabotropic glutamate receptor agonist (DCG-IV) reduces the frequency of mEPSCs and SPWs. ***A***, mEPSCs collected from field CA3b prior to and after an infusion of DCG-IV (2 µM). ***B***, DCG-IV caused a mark reduction in the frequency of high voltage SPWs and an increase in low voltage, fast activity. These effects reversed upon drug wash-out. ***C***, A 30 min infusion of the agonist caused a rapid and reversible reduction (57%) in EEG power in the SPW frequency range (p<0.001, repeated measures ANOVA; n = 4 slices).

DCG-IV [(2S,2′R,3′R)-2-(2′,3′-dicarboxycyclopropyl)glycine], an agonist for the type 2/3 metabotropic glutamate receptors (mGluR 2/3) is reported to depress mossy fiber, but not CA3 commissural-associational, transmission by inhibiting release [40]. The agonist (2 µM; 20 min) reduced the mean frequency of mEPSCs recorded from CA3b neurons with the greatest effect, as expected, occurring on larger events; mEPSCs below 20 pA decreased by 33±19% while those lying between 20 and 50 pA were reduced by 53±19% (p = 0.0008, 2-tailed paired t-test; n = 4 cells from 4 slices). DCG-IV at the concentration used in the above experiments (2 µM) increased low voltage, high frequency activity, in accord with earlier work [41], while lowering the incidence of the high voltage SPWs (**Fig. 6A**). Power in the SPW frequency range decreased by 57±15% (p<0.01; repeated measures ANOVA), and this effect reversed upon washout (**Fig. 6B,C**). These results provide the first evidence that increases and decreases in constitutive release are accompanied by corresponding changes in SPW activity. While the effects could reflect contributions from non-mossy fiber sources, the differential actions of DCG-IV on large mEPSCs agrees with prior reports [40] that the compound acts selectively on mossy terminals in CA3.

### Factors responsible for the intervals between SPWs: Tests for contributions from slow hyperpolarization

The above studies point to the conclusion that mossy fiber synapses initiate SPWs but leave open the question of why individual waves are separated by long, irregular intervals. SPWs are accompanied by strong IPSCs [15], [42], presumably generated by local feedforward and feedback connections. While these inhibitory events would be expected to reduce the probability of discharges and thus the production of population events, fast IPSCs are too brief to account for the >200 ms delays that typically separate SPWs. The various slow hyperpolarizing potentials that can follow large EPSPs provide a more likely means for establishing long intervals between SPWs. However, large, long-lasting positive-going events are not evident in intracellular recordings of the waves and, as described below, agents that block slow hyperpolarizations do not increase SPW frequency.

**Figure 7:**
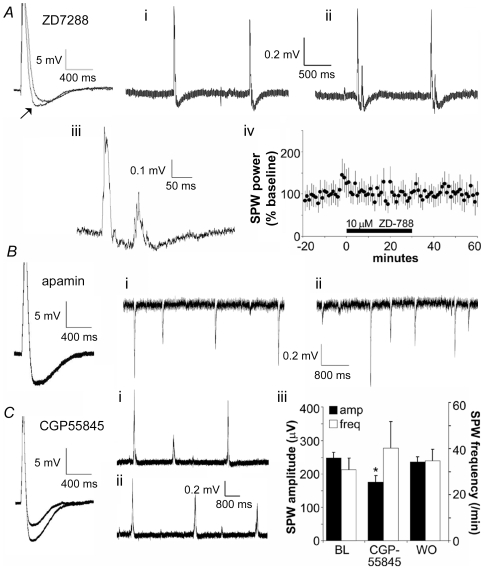
Effects of manipulating after-hyperpolarizing potentials (AHPs) on SPWs. ***A***, ZD7288 (10 µM), an antagonist of *I*(h), reduces the AHP (arrow) found immediately after the composite EPSC produced by a brief burst of stimulation pulses, but has little effect on the later component of the AHP in CA3 neurons. Experiment was repeated in 3 cells from 3 slices. (i) SPWs recorded 10 minutes before and (ii) 10 minutes after the infusion of 10 µM ZD7288. Note the presence of a single small potential following each SPW under the latter conditions. (iii) Enlargement of example trace from (ii) indicates that the after-discharges have a similar waveform to SPWs. (iv) Power of the EEG spectrum (% baseline [bl]) in the SPW frequency range is unaffected by ZD7288 (n = 4 slices). ***B***, Apamin (100 nM, 20 min), an inhibitor of SK channels, does not affect the composite EPSC elicited by a brief burst of afferent stimulation (left panel). Effect representative of 3 cells tested. Frequency and size of SPWs collected before (i) and during (ii) 100 nM apamin infusion are comparable. Records were collected from the apical dendrites of field CA3 and illustrate the reversed polarity associated with a local dipole. ***C***, CGP55845 (50 µM, 20 min), an antagonist of GABA-B receptors, reduces the AHP elicited by an intense burst of afferent stimulation (left panel). Effect representative of 4 cells tested. SPWs observed 10 min prior to (i) and during (ii) 50 µM CGP55845 infusion did not show detectable differences in frequency or shape. (iii) The GABA-B antagonist produced a small depression of SPW amplitude (*p<0.05, paired t-test; n = 3) but did not reliably increase the frequency (p>0.3) of the waves. (BL:baseline; WO: washout).

Recent work suggests that the *I*(h) (hyper-polarization activated cation current) is a principal source of the AHP that follows spike-free EPSCs elicited by stimulation of the Schaffer-collateral projections in hippocampal slices [42]. Consonant with this, the *I*(h) channel blocker ZD7288 at 10 µM [43], the half-maximal inhibitory concentration in CA1 [44], reduced the initial segment of the AHP recorded from CA3 neurons (recorded at −70 mV) that follows the composite EPSC elicited by a burst of afferent stimulation (**Fig. 7A**, arrow). ZD7288 hyperpolarized the pyramidal neurons by 5–7 mV, presumably because a significant number of *I*(h) channels are open under resting conditions. Despite its potent effects on mid-duration AHPs, ZD7288 did not change the mean frequency of the full-sized SPWs, although it did increase the likelihood that individual SPWs would be followed by 1–2 smaller waves (**Fig. 7A** **, i–iv**).

Calcium-dependent potassium channels are known to underlie much of the AHP that follows action potentials in hippocampal neurons. Blocking these potentials by loading clamp electrodes with the calcium-chelating agent BAPTA had no detectable effect on intracellularly recorded correlates of SPWs (data not shown). Moreover, inhibiting SK-type calcium-activated potassium channels, which in many cells mediate 50–200 ms AHPs that follow intense cell spiking [45], [46], throughout the slice with the selective toxin apamin had little if any effect on SPW frequency in 3 slices tested (**Fig. 7B** **, i,ii**).

Finally, there is the possibility that the long-lasting hyperpolarization produced by synaptic activation of GABA-B receptors in hippocampus [42], [47] contributes to the spacing of SPWs. The selective antagonist CGP55845 had only minor effects at normal stimulation intensity but produced a sizable suppression of the hyperpolarization elicited by supra-maximal intensity (**Fig. 7C**). These results are in agreement with those from earlier reports [42]. CGP55845 did not detectably modify the frequency of the waves, although it caused a modest yet significant decrease in their amplitude (**Fig. 7C** **, i–iii**). A previous study found that the compound does not affect the voltage dependency of SPWs in field CA1 [15].

### Factors responsible for the intervals between SPWs: Role of mEPSC frequency

**Figure 8:**
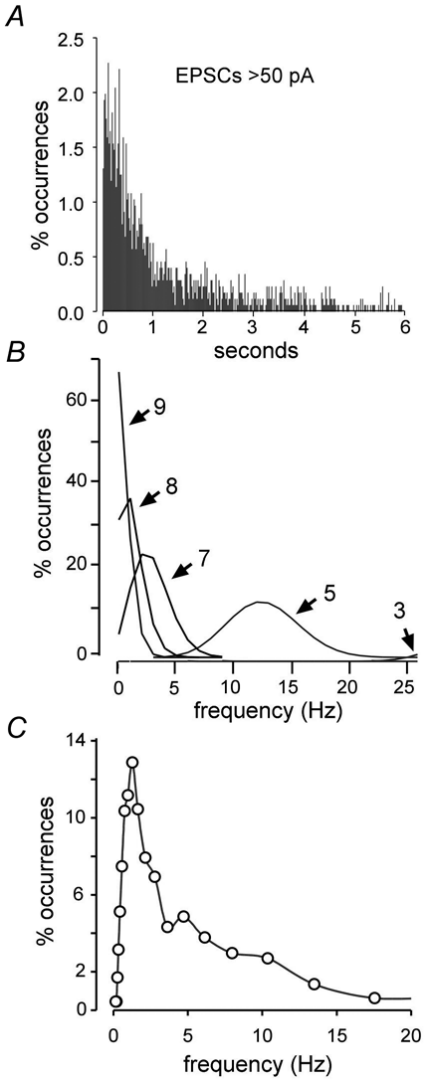
Frequency at which small collections of simulated neurons fire within the same 20 ms time bin in response to inputs arriving at the observed temporal distribution of large mEPSCs. ***A***, Interval histogram for mEPSCs greater than 50 pA recorded from nine CA3b pyramidal neurons. ***B***, Frequency at which the indicated numbers of neurons (arrow), from a simulation of 500 cells, fire during the same 20 ms time bin. Each cell in the model received the input shown in panel A. ***C***, Frequency distribution of SPWs recorded from field CA3b under baseline conditions.

An alternative to the idea that long hyperpolarizations provide the temporal spacing for SPWs is that the intervals are defined by the probability of the initiating events. Technical considerations, including the use of TTX for recording mEPSPs, preclude direct testing of relationships between SPW frequency and the parameters of spontaneous release. Accordingly, a small simulation was used to investigate the question of whether large (≥50 pA) mEPSCs will co-occur on a subpopulation of pyramidal cells with sufficient frequency to account for the observed frequency of SPWs. The simulation consisted of n objects that were programmed to emit events stochastically, but with the same inter-event frequency distribution as was recorded from CA3b pyramidal neurons (n = 9 cells from 9 slices). The distribution was similar to those obtained in previous reports [30], [33]. Measured time intervals between mEPSC events were analyzed, and it was determined that their probability distribution was well modeled with a power law (**Fig. 8A**) as follows: 
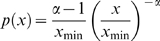
(1)where x is the time until the next mEPSC event. The exponent was determined to be 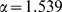, via the equation
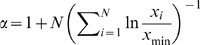
(2)where *N* is the number of data points [48].

Simulations were performed to determine the probability distributions of the number of simultaneous firings, in bins of 20 ms, given M cells emitting events according to equation 1. To perform the simulation, the value of M was fixed (i.e. M = 100) and a buffer buff(i) was initialized to zero where i corresponds to a 20 ms bin and i ranges over the length of the simulation. The main loop of the simulation is then
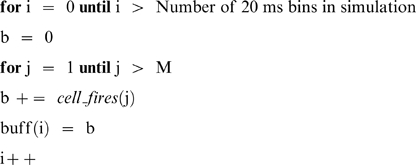
The routine *cell_fires* returns a 1 if cell j fires at time i and zero otherwise. A probability distribution was determined from the above by looping over buff, counting the number of simultaneous firings (k = 0,1,2,3,…) where, for example, a value of 24 for k = 6 means that 6 cells were found to fire simultaneously 24 times in buff (i.e., during the full run of the simulation). We then divided these values by the size of the array to yield probability values.

The routine *cell_fires* was implemented as follows. At the beginning of the simulation, cell data structures, one for each cell in the simulation, are initialized with a “time until firing” value, where this value is set stochastically according to the power law distribution above. Then, for each call to *cell_fires*, which corresponds to a time increment of 20 ms, cell j is checked to see if the time i indicates that it is time to fire. If it is, then a new time is set for the next firing, and the routine returns a 1. Otherwise, the routine simply records that the time until firing for cell j has just been decremented by 20 ms.

The output of the simulation was the probability distribution of such simultaneous firings over the length of the simulation, which was allowed to run until the distribution converged. This led to estimates of frequency distributions for the near simultaneous discharge of ≥ n cells. The simulation output probability distributions (of frequencies at which ≥ M cells fire together) were Poisson, i.e., they followed the form
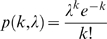
where we determined that *λ* depends on M, the number of cells in the simulation. Hence, by mathematical modeling and stochastic simulation, we established that 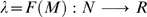
and therefore obviates the need for further simulations.

From the above, we determined that conjoint activity of 7–8 (out of 500) units occurred at about the same mean frequency as SPWs (**Fig. 8B**); however, the distribution of recorded SPW frequencies (**Fig. 8C**) was skewed toward higher frequencies to a greater degree than was found in the simulations. In all, if near simultaneous firing of approximately 2% of the neuronal population is sufficient to initiate a propagating event within field CA3, then the frequency of large mEPSCs occurring on those cells could account for much of the frequency distribution associated with SPWs.

**Figure 9:**
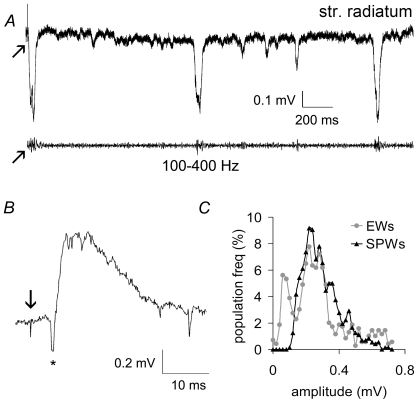
Minimal stimulation of the mossy fibers triggers CA3 sharp waves. ***A***, Stimulation pulses (arrows) were delivered to the infragranular zone of the hilus to activate the mossy fibers arising from a small portion of the granule cell population. This reliably triggered a negative-going, complex response in the apical dendrites (str. radiatum) of field CA3b; the size and shape of this response closely resembled those of spontaneous SPWs. High-pass filtered trace (100–400 Hz; time-locked to upper trace) showed that both the evoked response and spontaneous SPWs were accompanied by high frequency activity, particularly on their ascending phases. ***B***, As with SPWs, the complex potentials elicited by mossy fiber stimulation were positive in sign when recorded at the boundary between str. lucidum and str. pyramidale. Note that a small mossy fiber response (asterisk) precedes the positive wave. The downward arrow denotes the stimulation pulse. ***C***, The frequency distribution for the peak amplitude of the evoked waves (EWs) illustrates the highly variable nature of the responses over the course of a recording session. Note the extensive overlap for the evoked and spontaneous wave distributions.

If co-occurrence of large, mossy fiber mEPSCs can trigger a SPW, then minimal activation of the projections should have a comparable effect. Prior work suggests that this is the case. Specifically, spontaneous discharges of a sub-population of granule cells (‘dentate spikes’) are routinely followed by a sharp wave in field CA3 [19]. Explicit tests of the point were made here by asking if stimulation of a small population of mossy fibers generates a CA3 population event with the characteristics of a SPW. Stimulation pulses (3/min) were delivered to the infragranular zone near the tip of the internal wing of the dentate gyrus (Fig. 2A). Use of this site restricts activation to the fibers emerging from a small percentage of the total granule cell population. Single pulses reliably triggered a single negative-going, complex wave in the str. radiatum of field CA3b with a 3–4 ms delay in each of four slices. The responses occurred in isolation without after-potentials and had no evident effect on spontaneous SPWs (**Fig. 9A**). They were accompanied by high frequency, (100–400 Hz) ripple-like activity that was particularly evident on the rising phase of the potential, as was also the case for SPWs (Fig. 9A, top trace). The laminar profile for the evoked waves – negative in the dendrites, positive in the cell body layer (**Fig. 9B**) – also closely resembled that for SPWs. Importantly, the amplitude of the evoked waves varied substantially and unpredictably across stimulation pulses. The frequency distributions of peak amplitudes for the evoked and spontaneous waves were comparable (**Fig. 9C**). Stimulation of the commissural-associational projections did not elicit responses of the type just described. The field potentials were larger, shorter in duration, and lacked the complexity of those evoked by mossy fiber pulses; moreover, they had constant amplitudes and were not accompanied by low voltage, high frequency activity.

Finally, recent evidence suggests the existence of gap junctions between mossy fibers [49]. In light of the model proposed here, these may amplify weak signals and thus establish a threshold for SPW generation. As previously reported [15], the gap junction blocker carbenoxolone (100 µM) reduced both SPW frequency and SPW mean amplitude (46±6% of baseline; p<0.01, paired t-test; n = 4 slices). Minimally evoked mossy fiber responses elicited in the same slices exhibited a parallel reduction in amplitude (43±11%; p = 0.02, n = 4). These results accord with the hypothesis that SPWs originate with events occurring at mossy fiber synapses.

## Discussion

Various results indicate that sharp waves propagate between hippocampal subfields along associational projections [7], [16], [19], but the present results constitute the first evidence concerning the factors that initiate individual waves. Cutting the perforant path or mossy fibers did not eliminate the SPWs, although the latter manipulation reduced their amplitude and slightly decreased their rate of occurrence. These observations rule out the possibility that waves are driven by spontaneous activity in extrinsic structures projecting to field CA3. The mossy fiber result does suggest that spontaneous dentate events (i.e., DWs) enhance ongoing SPW activity. Previous experiments showed that DWs precede, and thus presumably initiate, an individual, unusually large sharp wave by 2–5 ms [19]. However, the dentate waves are much less frequent than SPWs and thus cannot be responsible for most of the activity. Combined, the results suggest that spontaneous activity in the dentate periodically induces a sharp wave and thereby may modulate the amplitude of subsequent potentials in CA3.

Removal of the mossy fibers through destruction of the dentate gyrus almost eliminated SPWS. The CA3 commissural-associational system remained intact in these preparations, and the occasional waves observed in the denervated slices had normal amplitudes. Coupled with the transection results, this finding points to constitutive release from mossy fibers as the triggering event responsible for the large majority of SPWs. Consonant with this, DCG-IV, a type 2/3 mGluR agonist reported to selectively depress mossy fiber transmission [40], [50], [51], reduced the frequency of SPWs at concentrations that depressed the occurrence of large miniature EPSCs. Conversely, forskolin, which enhances mossy fiber transmission [39], increased the incidence of both mEPSCs and SPWs.

What is responsible for the long intervals between SPWs? Recent work indicates that the ripples that ride on the waves reflect the activity of local inhibitory interneurons [13], [14], while whole cell recordings have demonstrated that large IPSCs accompany SPWs [15], [19]. It is thus reasonable to conclude that inhibition dampens the likelihood of SPW initiation for a brief period after the occurrence of a wave. Other studies show that activation of NMDA-type glutamate receptors accompanies and markedly depresses the amplitude of the waves [42] but blocking the receptors did not substantially affect their incidence. Nor, as shown here, is their incidence affected by compounds that block medium-duration after-hyperpolarizing potentials (AHPs). These potentials are triggered by intense synaptic responses or cell firing and can serve to suppress future spiking for hundreds of milliseconds [52]. As within-cell events, AHPs would not depress groups of neurons, in the manner of feedback and feedforward inhibition, but they could cause deletions from the pool of cells available to participate in an initiating event and in this way reduce the probability (and thus frequency) of SPWs. A blocker of *I*(h) reduced the size of synaptically induced AHPs and caused SPWs to be followed by a set of 2–4 small after-potentials. It is thus likely that a combination of *I*(h) generated hyperpolarization and GABA-A dependent inhibition depresses excitability after the appearance of a SPW and thereby prevents reverberating activity. Despite these effects, the antagonist did not significantly modify the mean frequency of fully developed sharp waves. Apamin, a blocker of SK-type potassium channels that contribute to an ∼200 ms hyperpolarizing current, left frequency unchanged and produced little if any modification to the size of the SPWs. In all, it seems unlikely that the spacing between the waves is set by medium and slow AHPs.

In contrast to the above conclusion, work using thick hippocampal slices found that large slow AHPs follow individual SPWs and that suppression of these increases the frequency of the waves [53]. The difference between these results and those we obtained with conventional slices may arise from the larger CA3 associational networks presumably captured in the thicker preparations. That is, convergence by larger numbers of inter-connected neurons could result in deeper depolarization and greater cell spiking, with the latter event triggering the calcium-dependent potassium conductances underlying the slow AHP. Alternatively, it is possible that metabolic differences between the two slice preparations, or between interface and submerged slices, affected the likelihood of triggering slow after-potentials. In any case, our results strongly suggest that the minimum conditions needed to generate appropriately spaced SPWs do not include AHPs.

An alternative explanation for the long and highly variable intervals between SPWs is that these factors reflect low probability events emerging from the quasi-random nature of spontaneous release. This could involve occasional occurrence of a very large mEPSC or of multiple mEPSCs from the mossy fibers, either of which are events that could trigger an action potential and thus contribute an element needed for the production of a sharp wave. If this were to happen at a sufficient number of neurons in the same time frame, then the non-linear, positive feedback system embedded in the dense CA3 associational system would recruit a substantial portion of the total neuronal population. Work with a small model suggested that this is not an unlikely explanation for the spacing of SPWs. It is noteworthy in this context that the mossy fibers innervate interneurons in field CA3 and thereby generate potent feedfoward inhibition onto the pyramidal cells [54], [55]. This will serve to dampen the excitability of the latter neurons and reduce the likelihood of sharp wave formation; quasi-random variations in di-synaptic inhibition would generate local differences in the threshold mEPSP size needed to excite a given pyramidal cell. While these points do not affect the conclusions drawn from the minimal model, which deals with average conditions, they do suggest that plasticity will influence the incidence of SPWs. Specifically, repetitive mossy fiber activity causes a pronounced facilitation of release onto the pyramidal neurons [56], [57] and a depression of feedforward inhibition [54]. The present model predicts that these events will result in an increased frequency of SPWs. It will be of interest in future studies to determine if mossy fiber potentiation produces the expected changes.

The constitutive release argument predicts that evoked activity in the mossy fibers will trigger a sharp wave. Evidence supporting this is found in reports that a dentate spike, the spontaneous discharge of a sizeable population of granule cells, is reliably followed after an appropriate delay by a CA3 SPW [19]. Moreover, the present studies demonstrated that minimal stimulation of the mossy fibers triggers a large, complex response with multiple characteristics of SPWs (size, duration, ripples, and laminar profile). The evoked SPWs were highly variable with about the same frequency distribution of amplitudes as their spontaneous counterparts. Stimulation of the commissural-associational projections that terminate immediately distal to the mossy fibers triggered large field EPSPs of constant size but produced no sign of sharp waves. These results demonstrate that near-synchronous release from small numbers of mossy fiber terminals initiate reverberating responses and that other inputs are much less effective in this regard.

Agents that uncouple gap junctions reduced the likelihood of eliciting a sharp wave, in accord with earlier reports [15]. This could indicate that recently described gap junctions between mossy fibers [49] serve to amplify the effects of single stimulation pulses by recruiting unstimulated axons and thereby increase the likelihood of engaging enough pyramidal neurons to initiate a wave. Alternatively, there is evidence for electrotonic coupling between pyramidal cell axons and this would be expected to enhance the recurrent activity triggered by mossy fiber-driven activation of CA3 neurons.

The constitutive release hypothesis, in addition to providing an explanation for the origins of a characteristic feature of hippocampus, provides some insight into the functional significance of certain unusual features of the dentate gyrus/mossy fiber system. Granule cells have a thin, poorly myelinated axon that generates a small population of enormous terminals [58], [59]. The terminal boutons are also unusual in that they are densely packed with vesicles and make multiple synaptic contacts with the post-synaptic cell [60]. Striking as these features are, there has been surprising little speculation regarding their significance for the operation of the hippocampus. The hypothesis presented here is consonant with an anatomical system that has a high release probability (because of vesicle numbers and multiple contacts) and is capable of generating unusually large miniature EPSCs. These constitutive factors produce the variations in the excitability and spontaneous firing of the pyramidal cell targets needed to periodically recruit cells into population responses. The mossy fibers, in this view, not only serve to connect granule cells, and their cortical input, to field CA3 but also act as isolated elements with unusual properties appropriate for initiating autonomous waves.

Part of the answer to the question of how the peculiar features of the mossy fibers relate to hippocampal operations may thus lie in the significance of SPWs. The irregularity of the waves argues against models in which they organize hippocampal activity so as to be synchronized with arriving afferents, a type of function often postulated for more rhythmic activity (e.g., theta, gamma). An alternative suggestion builds on the observation that the mean frequency of SPWs is similar to that of the low frequency afferent stimulation commonly used to reverse long-term potentiation (LTP) [61], [62] when applied immediately after its induction. In support of this, theta burst stimulation, delivered at magnitudes just above those required to induce robust LTP in slices that lack SPWs, produces little or no LTP in temporal hippocampal slices exhibiting SPWs [22]. Moreover, antagonists of the adenosine A1 receptor at concentrations that block LTP reversal by low frequency stimulation fully restored potentiation in temporal slices. It thus may be the case that the unusual properties of the mossy fibers result in a system that, in the absence of intervening circumstances, reverses recent potentiation and thereby prevents long-term storage of weakly encoded information.

## Materials and Methods

All procedures were conducted in accordance with the National Institutes of Health *Guide for the Care and Use of Laboratory Animals* and protocols approved by the Institutional Animal Care and Use Committee of the University of California at Irvine.

### Slice Preparation

Male Sprague-Dawley rats (Charles Riber Labs, Wilmington, MA) approximately 4 weeks of age were anesthesized via halothane (Sigma-Aldrich, St. Louis, MO) inhalation and sacrificed by decapitation. The whole brain was quickly removed and placed in icy, oxygenated artificial cerebrospinal fluid (ACSF). The brain was then dissected, and a tissue block containing the hippocampus was prepared. To prepare this block, the brain was placed ventral side down, and the most anterior portions of forebrain and cerebellum were cut off and discarded. A cut was made through the midline to separate the two hemispheres. For each hemisphere, a cut was made at the posterior end of the tissue block that was angled approximately 45 degrees away from the coronal plane in the anterior medial direction (i.e., 45 degrees clockwise from coronal plane for left hemisphere and 45° counterclockwise from coronal plane for right hemisphere). The face of the last angled cut was glued to the stage of a vibrating tissue slicer (Leica VT1000; Bannockburn, IL) such that slices were cut roughly perpendicular to the longitudinal axis of mid-temporal (ventral) hippocampus. Slices were taken from this region because CA3 associational axons terminate closer to their cells of origin in the temporal hippocampus than they do in septal (dorsal) hippocampus [25]. Thus, the CA3 recurrent collaterals that are important for SPW propagation are more likely to be preserved within slices from temporal hippocampus [15], [24]. Slices were cut at a thickness of 350 µm. ACSF for slice preparation was of the following composition (in mM): 124 NaCl, 3 KCl, 1.25 KH_2_PO_4_, 5 MgSO_4_, 3.4 CaCl_2_, 10 D-glucose, 26 NaHCO_3_.

### Electrophysiological Recording

Slices were immediately transferred to an interface recording chamber and allowed to recover for >1 h prior to the start of an experiment. Recording ACSF was of the same composition as described above, except that MgSO_4_ and CaCl_2_ concentrations were lowered to 1 and 3 mM, respectively. Oxygenated ACSF was infused at a rate of 60 ml/h; additionally, warmed and humidified 95% O_2_/5% CO_2_ was blown into the slice chamber. Slices were maintained at ∼32°C during both recovery and recording.

For recording of field potentials, a chloride-coated silver wire was placed within a pulled glass micropipette (∼5 MΩ resistance) filled with 2 M NaCl. Recording electrodes were placed in either CA3b str. pyramidale or str. radiatum. Field potentials were digitized at 10–20 kHz using an AC amplifier (A-M Systems Model 1700; Carlsborg, WA), and 6–20 sec samples were recorded every min using NAC 2.0 Neurodata Acquisition System (Theta Burst Corp., Irvine, CA). Baseline measures were established for 15–30 min before the introduction of drugs to the bath through a second infusion line. Stimulation electrodes consisted of twisted nicrome wires attached to a stimulus isolation unit (Grass Instruments). Square wave pulses were set at 30–50 µA×0.03–0.05 ms for eliciting CA3 field responses and 15–25 µA×0.1 ms for minimal stimulation of mossy fibers.

As in previous intracellular studies of SPWs [21], whole cell recordings were made with 3–5 MΩ recording pipettes filled with solution of the following composition (in mM): 130 Cs gluconate, 0.1 EGTA, 0.5 MgCl_2_, 10 HEPES, 2 ATP (pH 7.25, 285 mosM). Holding potentials were −90 mV, if not indicated otherwise. Currents were recorded with a patch amplifier (AxoPatch-200A, Axon Instruments, Burlingame, CA). Recordings were collected from submerged slices, prepared from 3–4 week old rats, perfused at 2–3 ml/min with a 32°C solution composed of the following (in mM): 124 NaCl, 3 KCl, 1.25 KH_2_PO_4_, 3.4 CaCl_2_, 2.5 MgSO_4_, 26 NaHCO_3_, and 10 D-glucose, pH 7.3 and oxygenated with 95% O_2_/5% CO_2_. Miniature EPSCs were recorded in the presence of 1 µM tetrodotoxin (Sigma; St. Louis, MO).

### Reagents

CNQX, colchicine, forskolin, and carbenoxolone were purchased from Sigma. DCG-IV, ZD7288, CGP55845 and DPCPX were obtained from Tocris Cookson (Ellisville, MO).

### Dentate gyrus lesion

Rats were anesthetized with ketamine hydrochloride (50 mg/kg) and xylazine (10 mg/kg), and colchicine was injected into the dentate gyrus using a Hamilton microsyringe under stereotaxic guidance (coordinates from Bregma: 6.4 mm posterior, 4.4 mm lateral with bite bar at −3; 0.25–0.5 µl of 17 mM colchicine in isotonic saline was injected at 5.4 and 4.6 mm ventral to brain surface). Animals were used for electrophysiological studies 5 to 10 days after colchicine placement. For lesion verification, 350 µm thick vibratome sections (adjacent to those used for electrophysiological analysis) were either directly fixed in 4% paraformaldehyde or immersed in 0.37% sodium sulfide and then fixed in paraformaldehyde. The fixed slices were then subsectioned on a freezing microtome at a thickness of 30 µm, and stained with cresyl violet (directly fixed tissue) or processed for Timm's stain for heavy metals [63] plus cresyl violet staining (sodium sulfide-treated tissue).

### Measurements

Results are reported as means ± SD and illustrated as means ± SEM. Spectral power was estimated using the Fast Fourier Transformation function in MATLAB (MathWorks, Natick, MA). For analysis of effects on SPW power across time, spectral power was averaged within a band from 0.1–7 Hz and normalized with respect to average power during a ten minute baseline. Amplitude was measured as the peak amplitude from sample mean baseline. Significance for SPW power, peak amplitude, and frequency measures following drug treatment or transection was evaluated by 2-tailed Student's t-test comparison of mean values from baseline (10 min prior to wash-in) and post-manipulation (20–30 min following wash-in or transection) periods. Population values represent number of slices unless otherwise indicated.

### SPW detection

Detection of individual SPWs was performed by estimating the second derivative *d2*(*t*) of the signal *v*(*t*) at time *k* using data points *v*(*k*) and adjoining data points *v*(*k* − *h*) and *v*(*k* + *h*): [*d2*(*k*)  =  −*2v*(*k*) + *v*(*k* − *h*) + *v*(*k* + *h*)], where h is a parameter that was set according to the approximate half-width of the waves. A threshold was then selected based on the second derivative value that most accurately detected the events (values greater than this threshold signified an event). Additionally, only potentials ≥50 µV in size and 20–120 ms in duration were included. Accuracy of this detection method was verified by visual examination. Detected SPWs were saved as 400 ms epochs that included the 200 ms prior to the maximum amplitude of the SPW and the 200 ms that followed the SPW peak.

### Time frequency analysis

The time-varying energy in 10 Hz wide frequency bands, from 10 to 300 Hz, was calculated for individual SPWs using a method described by Tallon-Baudry et al. (1997) [64]. The 400 ms long epochs generated using the SPW detection method described above were convoluted by a family of complex Morlet's wavelets *w*(*t*, *f*), one for each frequency, as a function of time:
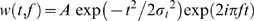
The coefficient *A* was set at:
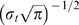
in order to normalize the wavelets such that their total energy was equal to 1. The family of wavelets was characterized by a constant ratio f/σ_f_, which was set to 7. Time frequency representations for multiple SPW events recorded from the same slice were then averaged.
